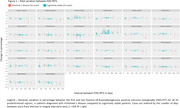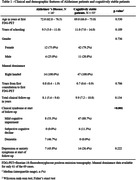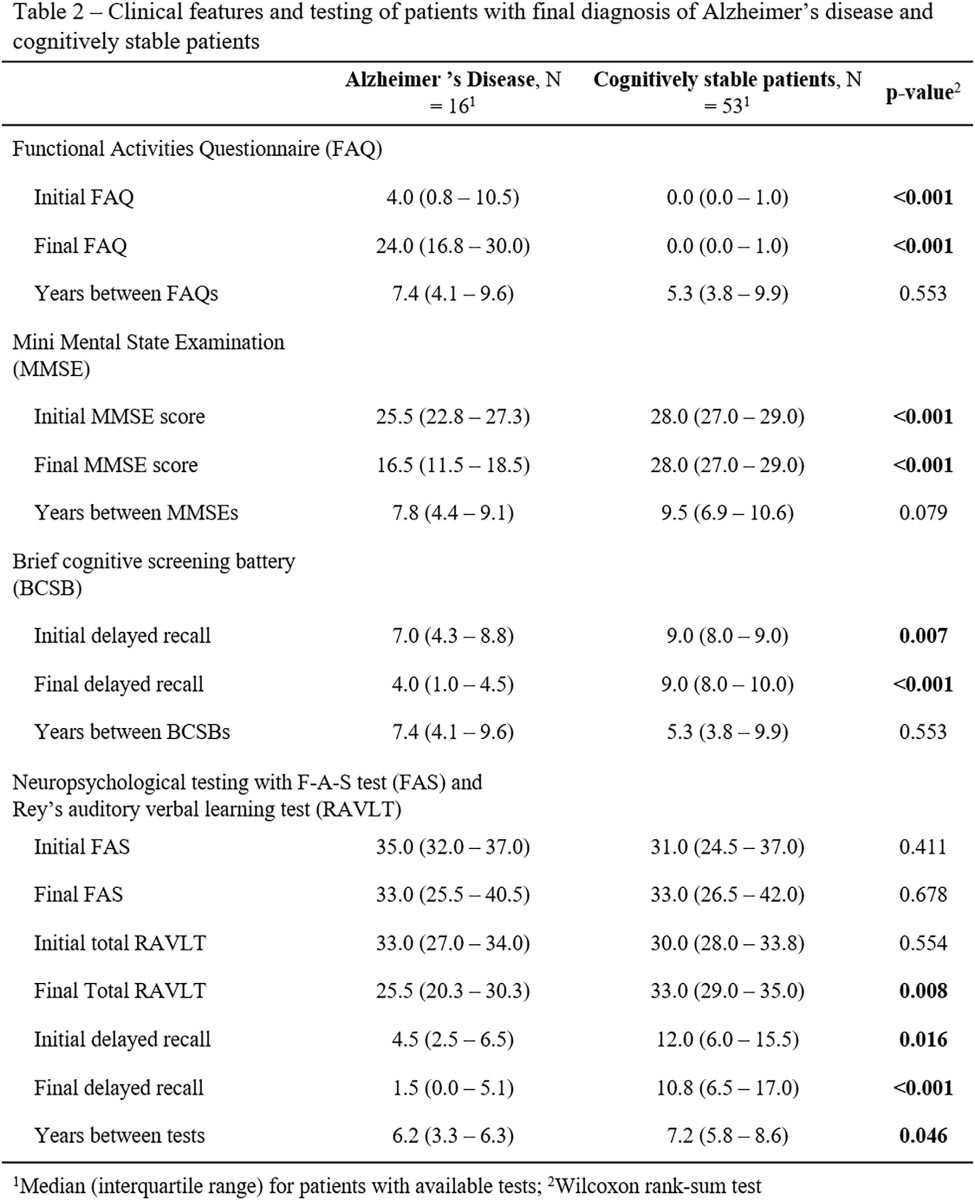# Measuring metabolic decrement with repeat FDG‐PET for the early diagnosis of neurodegenerative diseases

**DOI:** 10.1002/alz.093636

**Published:** 2025-01-09

**Authors:** Raphael de Luca e Tuma, Artur Martins Coutinho, Camila de Godoi Carneiro, Eduardo Sturzeneker Tres, Júlia Chartouni Rodrigues, Carlos Alberto Buchpiguel, Sonia Maria Dozzi Brucki

**Affiliations:** ^1^ Hospital das Clínicas, Faculdade de Medicina da Universidade de São Paulo, São Paulo, São Paulo Brazil; ^2^ Center of Nuclear Medicine, Institute of Radiology, Hospital das Clínicas, Faculdade de Medicina da Universidade de São Paulo, São Paulo, São Paulo Brazil

## Abstract

**Background:**

The diagnostic capability of fluorine‐18‐fluorodeoxyglucose positron emission tomography (FDG‐PET) is well established, but few studies have explored the utility of repeating scans, especially when using semi‐quantification methods to analyze the decrement between them.

**Methods:**

We retrospectively selected all patients at our clinic submitted to two or more FDG‐PETs. The initial and final FDG‐PET of each patient underwent semi‐quantitative analysis using the CortexID® Suite software (GE Healthcare) for 26 pre‐determined regions normalized for global cortical uptake. We subtracted the initial standardized uptake value ratio (SUVr) from the final SUVr to calculate the total variation. Patients with at least 3 years of clinical follow‐up were categorized based on their final diagnosis for comparison. Lastly, in patients with at least 1 year between exams, each total variation was then divided by the number of years between exams, to reach a value termed the “rate of metabolic decrement”.

**Results:**

We found 144 cases with two or more FDG‐PETs in our database, and 9 were excluded. There was a significant Pearson correlation coefficient between the total metabolic variation and the time between scans in 23 of the 26 regions. 103 cases could be clinically categorized: 16 were diagnosed with Alzheimer’s disease (AD) and 53 were cognitively stable (Table 1 & 2). All 26 regions were compared (Figure 1). Of note, analyzing only cases with at least 1 year between exams (83 cases), the posterior left cingulate of AD patients (14 patients) and cognitively stable cases (40 cases) had, respectively, an absolute variation of – 4.1% (IQR –5.6% ‐ –1.2%) versus +1.8 (IQR 0.0% ‐ +2.8%) and a rate of metabolic decrement per year of –1.1% (IQR –2.0% ‐ –0.6%) versus +0.4% (IQR 0.0% ‐ +0.7%), with a significant difference in both comparisons. 34 patients with other diagnoses (including 4 with behavioral‐variant frontotemporal dementia), were analyzed separately. 28 patients also had amyloid PETs available for analysis.

**Conclusions:**

Repeating an FDG‐PET may have a significant diagnostic benefit over a single scan. The rate of metabolic decrement could be a useful clinical tool for the early diagnosis of AD or in complex cases.